# Antitumor Mechanism and Therapeutic Potential of Cordycepin Derivatives

**DOI:** 10.3390/molecules29020483

**Published:** 2024-01-18

**Authors:** Linlin Cui, Li Zhao, Guanghuan Shen, Dahai Yu, Tian Yuan, Yingyu Zhang, Bo Yang

**Affiliations:** 1 College of Pharmacy, Harbin University of Commerce, Harbin 150076, China; 102638@hrbcu.edu.cn (L.C.); zhaol202312@163.com (L.Z.); shengh@hrbcu.edu.cn (G.S.); hrbadr@163.com (D.Y.); 17745602736@163.com (T.Y.); zyy2433280805@163.com (Y.Z.); 2 Heilongjiang Provincial Key Laboratory of Drug Prevention and Treatment for Senile Diseases, Harbin 150076, China

**Keywords:** cordycepin derivatives, antitumor, structural modification

## Abstract

Cordycepin has good antitumor activity, but its clinical application is limited due to the easy deamination of N6 in structure. In this study, a large lipolysis group was introduced at the cordycepin N6 to improve the problem, cordycepin derivatives (**3a**–**4c**) were synthesized, and biological evaluation of compounds was studied. In this study, the vitro antitumor activity of the compounds against MCF7 cells, HepG2 cells and SGC-7901 cells was evaluated by MTT assay. In the results, compound **4a** showed the most obvious inhibitory effect on MCF7 cells with an IC_50_ value of 27.57 ± 0.52 μM, which was much lower than cordycepin. Compound **4a** showed high selectivity between MCF7 and normal MCF-10A cells. Further biological evaluation showed that compound **4a** promoted apoptosis and blocked the cell cycle in the G0/G1 phase. Then, Western Blot was used to detect related apoptotic proteins. It was found that Compound **4a** could down-regulate the expression of Bcl-2 protein and up-regulate the expression of p53, Bax, Caspase-3 and Caspase-9 proteins. The mitochondrial membrane potential decreased continuously and the positive expression rate decreased. It was speculated that compound **4a** induced the apoptosis of MCF7 cells through the mitochondrial pathway.

## 1. Introduction

According to estimates from the World Health Organization, cancer incidence and mortality are rapidly increasing worldwide. Cancer is expected to rank as the leading cause of death in the 21st century. Despite the advances in cancer clinical research, a high percentage of oncological patients will suffer from overwhelming morbi-mortality.

*Cordyceps militaris* has been used in traditional Chinese medicine for a long time [1]. *C. militaris* exhibits a variety of clinical health effects including immunomodulatory, anticancer, antioxidant, anti-inflammatory and anti-microbial activities [2,3], and is applied as a functional ingredient in health foods and cosmetics [4,5]. In recent years, traditional Chinese medicine has been used to treat various cancers. Cordycepin is a nucleoside antibiotic, isolated and purified from C. militaris. Cordycepin has anti-inflammatory [6,7], antitumor [8,9,10], antiviral and immunomodulatory effects [11,12,13,14], and plays an important role in antitumor activity. Cancer is a major public health problem worldwide. It is the second leading cause of death after cardiovascular disease and the most important obstacle to further improving life expectancy. There is clear evidence that cordycepin inhibits breast cancer (caspase, MAPK), liver cancer (caspase, PLC) and stomach cancer (PI3K/Akt) through a variety of signaling pathways [15,16,17]. Studies have shown the antitumor mechanism of cordycepin; that is, cordycepin is phosphorylated after entering cells, and it is a monophosphate derivative, a diphosphate derivative or a triphosphate derivative. Triphosphate derivatives can inhibit mRNA synthesis and ultimately affect the synthesis of corresponding proteins, thus inhibiting tumor growth. At the same time, studies have shown that after cordycepin enters the body, rapid deamination occurs under the catalysis of adenosine deaminase (ADA) to produce the inactive metabolite 3’-deoxyinosine. This can lead to a short half-life of cordycepin in the body, and influence clinical treatment. If structural modification can effectively improve its susceptibility to deamination and short half-life, and effectively improve its bioavailability, it will become a feasible way to develop cordycepin drugs. Many studies on the modification of cordycepin mainly focus on the modification of hydroxyl and N_6_ positions. In this study, the hydroxyl group in cordycepin was modified, and heterocyclic rings were introduced in the N_6_ position. A series of cordycepin derivatives were designed according to the principle of bioelectron equiplatoon, and biological activities and mechanisms were evaluated through related experiments.

## 2. Results and Discussion

### 2.1. Chemistry

**Scheme 1:**
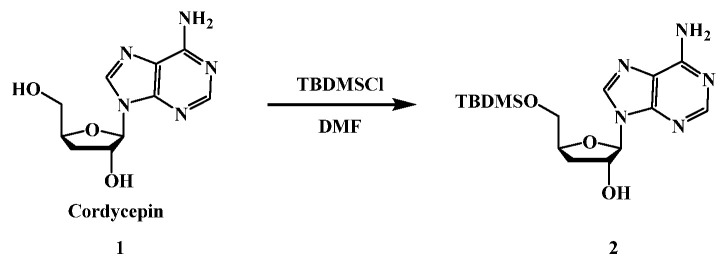
Synthesis of compound **2**.

**Scheme 2:**
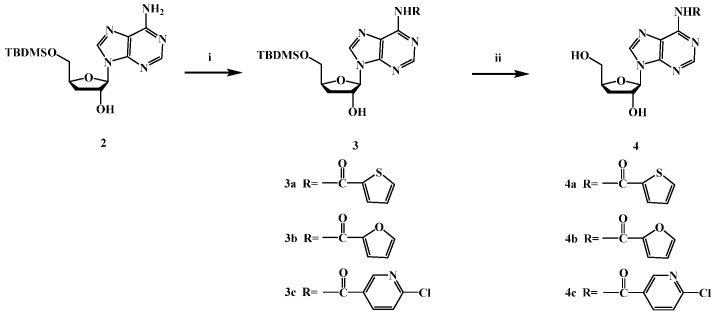
Synthesis of cordycepin derivatives (**3a**–**4c**). (**i**) RCI, pyridine, rt; (**ii**) TBAF, THF, rt.

The novel derivatives were synthesized according to the procedures shown in Scheme 1 and Scheme 2. As the substrate, cordycepin was connected to TBDMSCL at the 5’-OH position, which acts as an intermediate. Then, heterocyclic compounds were introduced at the N6 position, respectively. Target compounds **3a**–**3c** and **4a**–**4c** were synthesized and characterized by ^1^H-NMR, ^13^CNMR and HRMS.

### 2.2. Effects of Cordycepin Derivatives Inhibits the Proliferation on MCF7, HepG2 and SGC-7901

The assay of cell growth inhibitory effects of cordycepin derivatives **3a**–**4c** was tested against by MTT assay. The results are summarized in Table 1. The results show that compounds **3a**–**c** and **4a**–**c** inhibit cell proliferation in a concentration-dependent manner within the range of 0 to 80 µM. Compounds **3c**, **4a**, **4b** and **4c** have better inhibitory effects on MCF7 than cordycepin. For HepG2 proliferation inhibition, the effect of **4b** and **4c** is better. Compounds **3b** and **4a** show enhanced inhibitory effects on SGC-7901 compared with cordycepin. In the results, compound **4a** shows the most obvious inhibitory effect on MCF7 with an IC_50_ value of 27.57 ± 0.52. The results show that the modified compounds in the sugar ring had no obvious effect on the improvement of the activity, the introduction of heterocyclic rings in the base could improve the antitumor activity to a certain extent, and the introduction of thiophene ring had the best effect. It can be seen from the results that the structural modification of cordycepin can improve the antitumor activity effectively. Therefore, it is a feasible way to improve its activity by modifying its structure.

**Figure 1:**
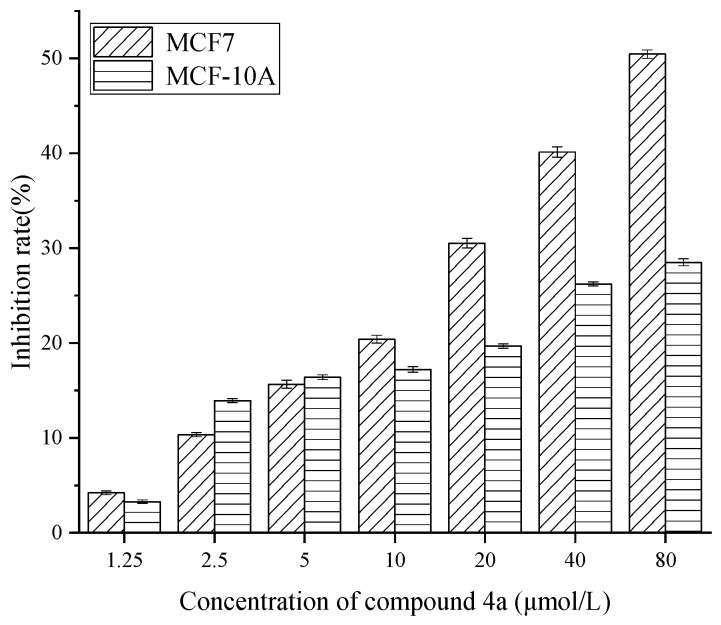
Inhibition rate of compound **4a** on MCF7 and MCF-10A cells.

Then, compound **4a** was evaluated for its in vitro cytotoxicity against one normal breast cell line (MCF-10A), which revealed low toxicity (Figure 1). With an inhibition rate ranging from 3.278% to 28.508%, compound **4a** has higher selectivity between MCF7 and MCF-10A. Therefore, this led us to select compound **4a** for further investigations against MCF7 breast cancer cells; according to its IC_50_ value, 56 μM, 28 μM and 14 μM were taken as the high, medium and low concentrations of compound 4a, HCPT (8 μM) and cordycepin (80 μM) were used as the positive control.

It has been reported that cordycepin significantly reduced breast cancer cell viability in a dose- and time-dependent manner and has low toxicity against normal cells but greater toxicity against breast cancer cells [18]. This reveals IC_50_ values ranging between 135 μM for MCF7 and 70 μM for MDA-MB-453 cells. In contrast to MCF7, cordycepin appeared to be preferentially active against highly de-differentiated breast cancer cells [19]. Previous studies have indicated that we performed experiments in an optimal non-toxic concentration (50 and 100 μM) of cordycepin with no change in morphology [20].

**Figure 2:**
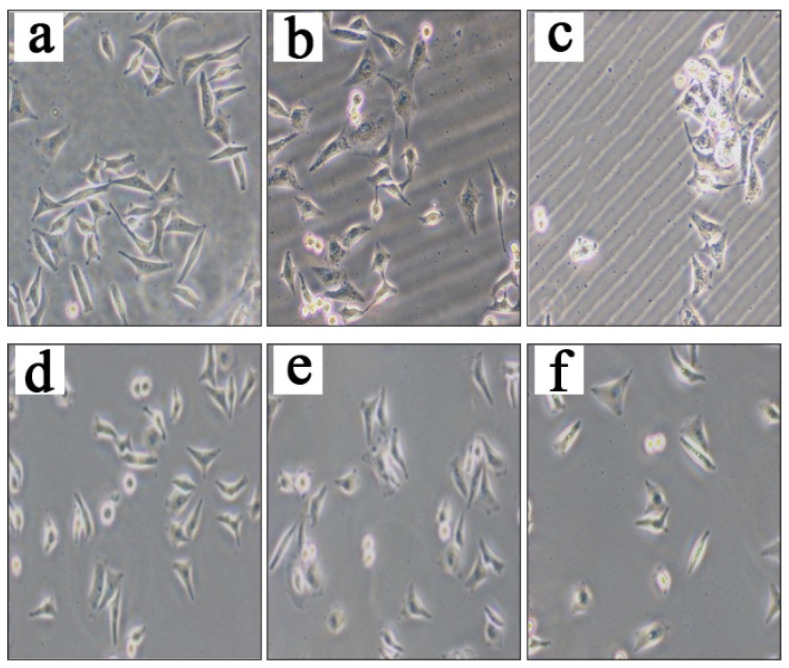
MCF7 cell morphology of the blank group (**a**), cordycepin group (**b**), positive control (**c**), low dose of compound **4a** (**d**), medium dose of compound **4a** (**e**), high dose of compound **4a** (**f**), (10 × 40 times).

Furthermore, inverted light microscopy showed a decrease in the number of MCF7 cells treated with compound **4a**. The fluorescence staining results are shown in Figure 2. Breast cancer MCF7 cells adhered to the wall growth, the cell outline was clear, the cell development was good, and the number was large. When treated with cordycepin (as shown in Figure 2b), compared with the blank group, the number of cells is reduced, the cell edges are not clear, and there are spherical dead cells. But when treated with the positive drug HCPT, as shown in Figure 2c, the number of cells is decreased significantly. We can find that cell morphology changed, and the dead cells are spherical.

Compared with normal breast cancer MCF7 cells, round dead cells can be seen when breast cancer MCF7 cells are treated with compound **4a** at the concentration of 14 μM in the low-dose group (as shown in Figure 2d), the number of cells in the low-dose group is reduced. The cell adherent state is damaged in the medium-dose group, and spherical dead cells could be seen. When treated with compound **4a** of 56 μM (as shown in Figure 2f), cells are not clear at the edge of the cells and had shrunken, and the number of cells is significantly less than that of the medium-dose cells, and spherical dead cells can be seen.

### 2.3. Compound ***4a*** Induces Apoptosis in MCF7 Cells

**Figure 3:**
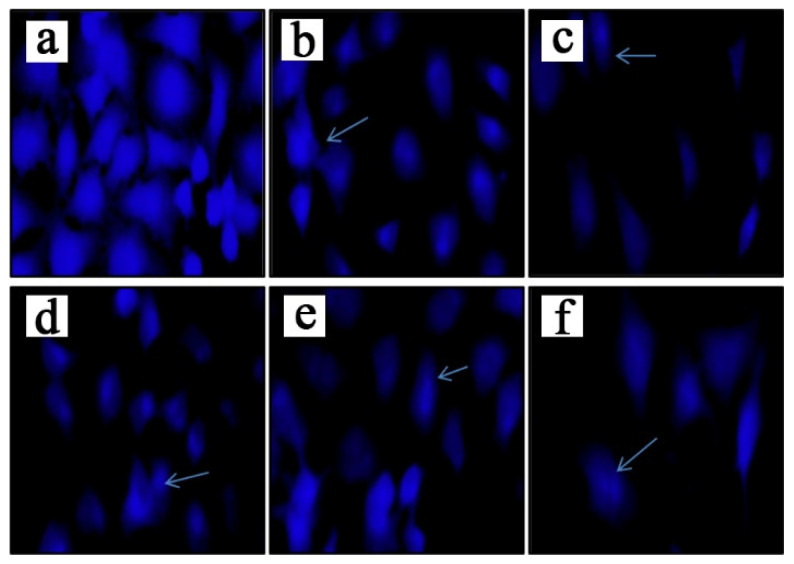
MCF7 cell morphology of the blank group (**a**), cordycepin group (**b**), positive control (**c**), low dose of compound **4a** (**d**), medium dose of compound **4a** (**e**), high dose of compound **4a** (**f**) under fluorescence microscopy (10 × 40 times).

Hoechst 33258 staining solution can pass through the cell membrane and combine with DNA inside the cell to produce blue fluorescence, thus reflecting the degree of apoptosis. Hoechst 33342 staining showed a significant increase in apoptosis in the MCF7 cells following compound **4a** treatment (Figure 3). As shown in the figure, after MCF7 cells were treated with the increase in the dose, the cell density showed a concentration trend and gradually decreased, the intracellular dense staining granular fluorescence was enhanced, the cell shrank, the adhesion was damaged, and the volume decreased. Compound **4a** broke the cell membranes leading to inducing the nuclear condensation by apoptotic in comparison with the control cells.

**Figure 4:**
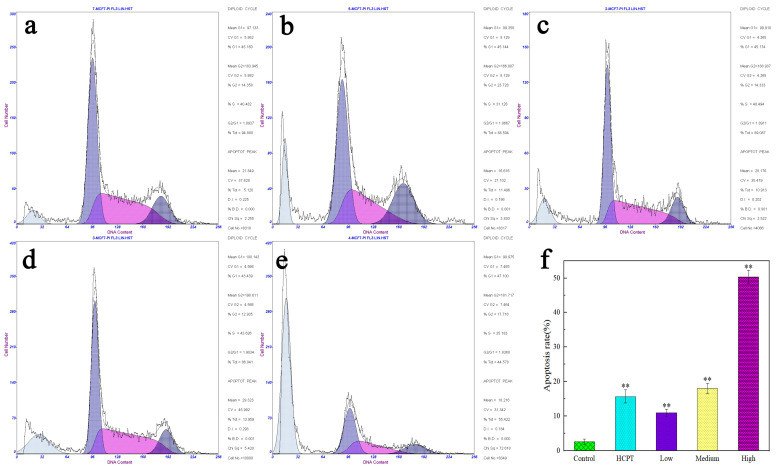
Flow cytometric detection of compound **4a** on the cycle of MCF7 cells (48 h). The blank group (**a**), positive control (**b**), low dose of compound **4a** (**c**), medium dose of compound **4a** (**d**), high dose of compound **4a** (**e**). Apoptosis rate (**f**); ($\bar{x}$ ± s, *n* = 3) compared with control: ** *p* < 0.01.

To further determine the basis of the anti-proliferative action of compound **4a** on the MCF7, we analyzed the cell cycle profile of the treated cells. Compound **4a** treatment at 14, 28 and 56 μM significantly increased the accumulation of G0/G1 cells. Compared with the control group, the percentage of MCF7 cells in the G1 phase significantly increased compared with that in the control group, respectively, indicating that compound **4a** induced MCF7 cell cycle arrest at the G0/G1 phase in a dose-dependent manner (Figure 4). We analyzed the proportion of apoptotic MCF7 cells by flow cytometry. After being treated with compound **4a**, notably, as the compound **4a** concentration increased, the degree of apoptosis also increased. As shown in Figure 4f, at 56 μM compound **4a**, the percentage of apoptotic cells reached 50.422 ± 1.862%. Therefore, these results demonstrate that compound **4a** exerts an apoptotic effect on MCF7 cells.

Previous studies have indicated that in the MCF7 cells, treatment with etoposide leads to shrinkage of both chromatin and whole cells, without the appearance of endonucleosomal cleavage of DNA Typical characteristics of apoptosis [21], including DNA fragmentation, were not evident in cordycepin-treated MCF7 cells [22]. Since apoptosis induction is the mode of action of most anti-cancer drugs [23], we also analyze the potential apoptotic effects of compound **4a** on the MCF7 in subsequent studies. Apoptosis can lead to increased permeability of the cell membrane [24], and previous studies have indicated that cordycepin administration promoted G2/M arrest and apoptosis of MCF7 cells [25]. It is known that inhibition of cell proliferation is often caused by cell cycle arrest [26].

**Figure 5:**
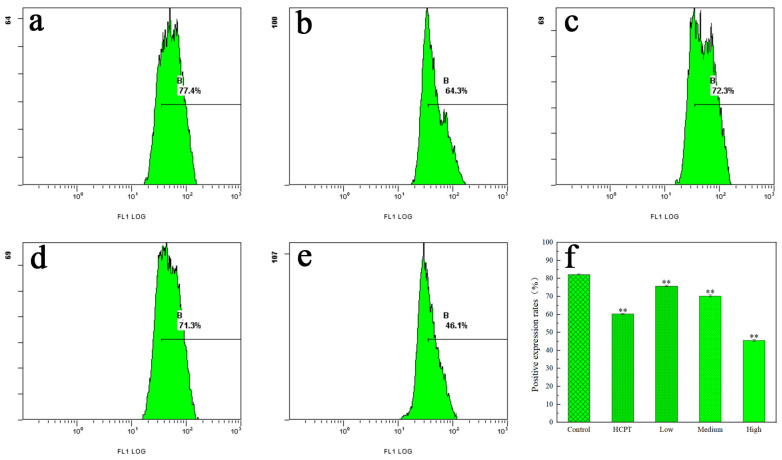
Effect of compound **4a** on mitochondrial membrane potential in MCF7 cells by flow cytometry (48 h). The blank group (**a**), positive control (**b**), low dose of compound **4a** (**c**), medium dose of compound **4a** (**d**), high dose of compound **4a** (**e**), positive expression rate (**f**); ($\bar{x}$ ± s, *n* = 3) compared with control: ** *p* < 0.01.

Additionally, to investigate the proapoptotic effect of compound **4a** on breast cancer period of time event, flow cytometry staining with Rhodamine 123 was used to detect the effect of compound **4a** on the mitochondrial membrane potential of MCF7. The effects of different doses of compounds on membrane potential are shown in Figure 5a–e. The results show that, with the increase in dose, the positive expression rate and cell membrane potential decrease gradually, and have a dose–effect relationship. The positive expression rates are shown in Figure 5f. The results are significant and indicate compound **4a** could significantly reduce MCF7 cells. Granular membrane potential induces apoptosis.

### 2.4. Results of Western Blot Analysis

**Figure 6:**
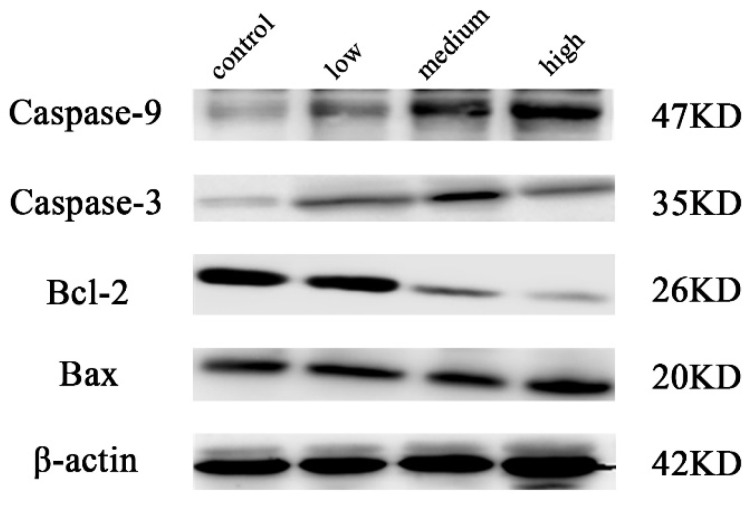
Western Blot method was used to detect the effect of sophocarpine on the expression of Caspase-9, Caspase-3, Bcl-2 and Bax in breast cancer MCF7 cells.

**Figure 7:**
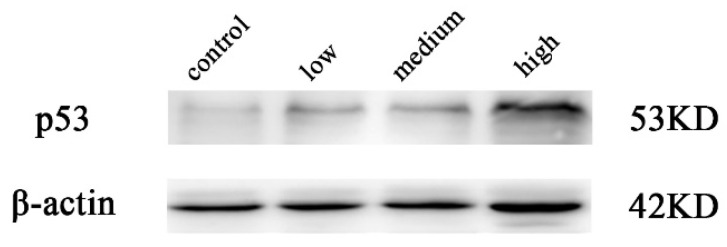
Western Blot method was used to detect the effect of sophocarpine on the expression of p53 in breast cancer MCF7 cells.

**Figure 8:**
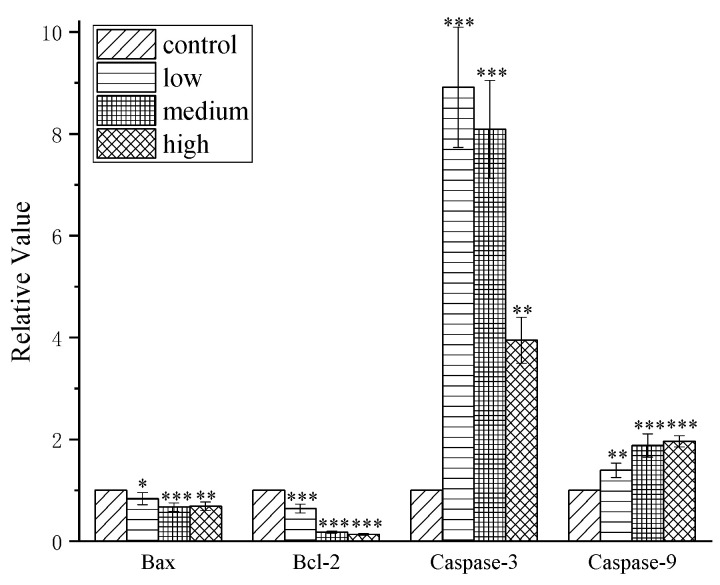
Western Blot detection of Bax, Bcl-2, Caspase-3 and Caspase-9 protein expression ($\bar{x}$ ± s, *n* = 3); compared with control: * *p* < 0.05, ** *p* < 0.01, *** *p* < 0.001.

**Figure 9:**
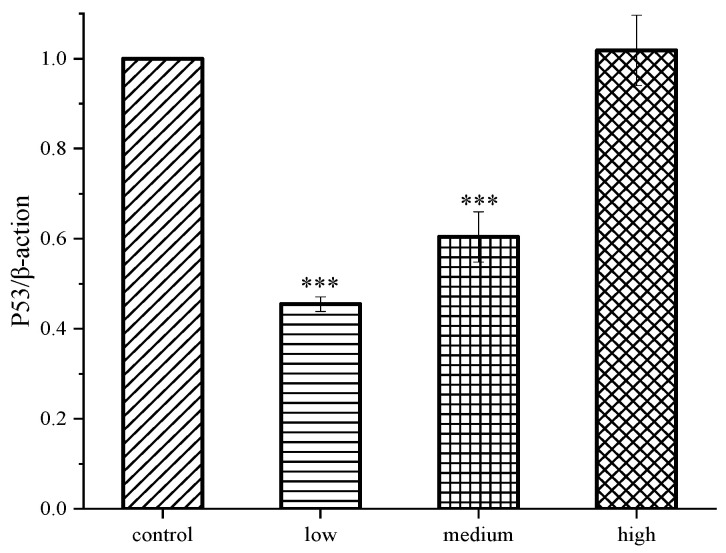
Western Blot detection of p53 protein expression ($\bar{x}$ ± s, *n* = 3); compared with control: *** *p* < 0.001.

To investigate the mechanism and induction of cordycepin on MCF7 cell activity, after different concentrations of compound **4a** on cells, test results of associated proteins by way of Western Blot assay were shown in Figure 6, Figure 7, Figure 8 and Figure 9. In this experiment, the blank group, compound **4a** low dose (14 μM), medium dose (28 μM) and high dose (56 μM), Western Blot detected the expression levels of related apoptotic proteins, Caspase-9, Caspase-3, Bcl-2, Bax and p53 in each group. The results showed that compound **4a** could down-regulate the expression of Bcl-2 protein and up-regulate the expression of Caspase-9, Caspase-3, Bax and p53.

There are three confirmed apoptosis pathways: death receptor pathway, mitochondrial pathway and endoplasmic reticulum pathway. After receiving apoptosis stimulation, cyt-C enters the cytoplasm through the mitochondrial membrane and activates caspase-9 and caspase-3. Caspase-3 is in the downstream effector part of various apoptotic pathways and acts as the ultimate executor of the caspase cascade reaction. The Bcl-2 family is an important regulatory protein in the mitochondrial pathway of apoptosis. The pro-apoptotic and anti-apoptotic proteins of the Bcl family remained relatively stable, but when Bax is overexpressed, the number of Bax/Bax homologous dimers increased, and the cell response to the death signal was enhanced, promoting the occurrence of apoptosis. However, when Bcl-2 is highly expressed, the activation of caspase-3 is inhibited, and more heterodimers of Bcl-2/Bax with stable structure are formed, which can resist the induction effect of Bax/Bax on apoptosis. The ratio relationship between Bcl-2 and Bax is critical for cell survival. And p53 functions beyond its ability to trigger cell cycle arrest and programmed cell death and new activities such as regulating metabolism, autophagy and cellular oxidation status. P53 protein can specifically inhibit the expression of Bcl-2, secrete pro-apoptotic proteins (Bax, Bad, Bak) and anti-apoptotic proteins (Bcl-2, Bcl-xl) to induce the activation of pre-apoptotic protein Bax, induce mitochondria to release cyt-C and form apoptotic bodies. Activate the Caspases (Caspase-3, Caspase-9) pathway and cause cell apoptosis through protease hydrolysis. In this experiment, with the increasing dose of compound **4a**, the relative expression levels of BAX, caspase-3 and p53 showed an increasing trend, while the expression of Bcl-2 showed an obvious decreasing trend, which indicated that compound **4a** could promote apoptosis of MCF7, and the promotion effect of high dose on apoptosis was more obvious than that of low dose. We suggest that compound **4a** may induce apoptosis of MCF7 in breast cancer through the mitochondrial pathway.

## 3. Conclusions

We modified the structure of cordycepin and synthesized new cordycepin derivatives by introducing fat-soluble groups on the N6 groups. The MTT method was used to investigate the bioactivity of the synthesized compounds against MCF7 cells of breast cancer, HepG2 cells of liver cancer and SGC-7901 cells of gastric cancer. Compound **4a** showed significant inhibitory activity against MCF7 cells with an IC_50_ value of 27.57 ± 0.52 μM, which was superior to cordycepin (46.85± 1.62 μM). In the cytotoxicity test, it was found to be less toxic to normal cells MCF-10A, showing high selectivity between breast cancer cells and normal cell lines. Therefore, compound **4a** was further studied to explore the effect of compound **4a** on MCF7 through in vitro cell experiments. The results showed that the number of breast cancer MCF7 cells decreased gradually in a concentration-dependent manner after treatment and spherical dead cells were visible. Compound **4a** can induce apoptosis and arrest the cell cycle in the G0/G1 phase. The related animal experiments need further verification.

In order to clarify the mechanism of compound **4a** affecting MCF7 cells, Western Blot analysis showed that compound **4a** could down-regulate the expression of Bcl-2 protein and up-regulate the expression of p53, Bax, Caspase-3 and Caspase-9 proteins. The mitochondrial membrane potential decreased continuously and the positive expression rate decreased. It was speculated that compound **4a** induced the apoptosis of MCF7 cells through the mitochondrial pathway.

## 4. Experimental Section

### 4.1. Chemicals and Reagents

Cordycepin (more than 99% in purity) (TIANBAO Biopharmaceuticals, Canton, China). 2-Thiophenecarbonyl chloride; 2-Furoyl chloride; 6-Chloronicotinoyl chloride, tert-Butyldimethyl chlorosilane (TBDMSCl), imidazole, and tetrabutylammonium fluoride (TBAF) were purchased from Aladdin (Shanghai, China). All reagents were of analytical grade. RPMI-1640 (Gibco, Oakland, CA, USA); fetal bovine serum (Sijiqing, Hangzhou, China); MTT (Solarbio, Beijing, China); HCPT (Shengtai, Harbin, China); DMSO (BASF, Shanghai, China). Mitochondrial membrane potential assay kit, cell cycle apoptosis analysis kit, trypsin, RIPA lysis buffer, and Hoechst 33258 were purchased from Beyotime (Shanghai, China); Infinite F50 Microplate reader (TECAN group, Männedorf, Switzerland); HF90 Incubator (Lishen, Shanghai, China).

### 4.2. Synthesis

#### 4.2.1. Synthesis of Compound **2**

Cordycepin (**1**) (1.5 g, 6 mmol) and 0.4446 g imidazole (680 mg, 10 mmol) were dissolved in dry DMF (6 mL) under ultrasound-assisted dissolution. TBDMSCl (1.5 g, 10 mmol) was added in batches. The reaction was stirred for 5 h at room temperature and then concentrated in a vacuum. The organic layer was washed with water and ethyl acetate, then afterward dried and concentrated, and then the crude product was purified by silica gel column chromatography with MeOH/CH_2_CI_2_ (1:15). The compound **2** was obtained.

#### 4.2.2. Synthesis of Compounds **3a**–**3c** (i)

Compound **2** (0.5 g, 1 mmol) was dissolved in dry pyridine (14 mL). 2-Thiophenecarbonyl chloride (0.57 g, 4 mmol), 2-Furoyl chloride (0.59 g, 4 mmol) and 6-Chloronicotinoyl chloride (1.13 g, 4 mmol) were added to the solution under N_2_ protection, respectively. The reaction was stirred at room temperature for 8 h and then cooled to 0 °C. NH_4_OH (28% aq) (4 mL) was slowly added to the solution. After the mixture was stirred for 30 min, the solvent was removed, and the residue was dissolved in EtOAc. The organic layer was washed with water, NaHCO_3_ solution and salt water. Organic layers were incorporated together, and then the solvent was removed by vacuum concentration. The residues were purified by silica gel column (MeOH/CH_2_Cl_2_ = 1:25) to obtain target compounds **3a**, **3b** and **3c**, respectively. The products were dried and weighed.

#### 4.2.3. Synthesis of Compound **4a**–**4c** (ii)

Compounds **3a** (0.43 g, 0.4 mmol), **3b** (0.42 g, 0.4 mmol) and **3c** (0.6 g, 0.4 mmol) were dissolved in THF (13 mL) and TBAF (0.94 mL, 1.0 M THF solution) at 0 °C respectively. The reaction was stirred at room temperature for 5 h, and then the solvent was removed. The residues were purified by silica gel column (MeOH/CH_2_Cl_2_ = 1:20) to obtain compounds **4a**, **4b** and **4c** respectively. The products were dried and weighed.

Melting points, optical rotation, TLC, ^1^H NMR. ^13^C NMR and MS were used to identify structures of new compounds. Melting points were tested on a YRT-3 melting point apparatus (Tianjin, China). Optical rotation was tested on PRO/P850 Polarimeter (Jinan, China). ^1^H NMR and ^13^C NMR were tested with Bruker Avance-500 MHz spectrometer (^1^H: 500 MHz, ^13^C: 125 MHz). Molecular weight was tested with TripIeTOF6600 (AB Sciex, Framingham, MA, USA).

### 4.3. Characterization

#### 4.3.1. 5′-O-(Tert-butyldimethylsilyl)-3′-deoxyadenosine (**2**)

^1^H-NMR (500 MHz, CDCl_3_) δ: δ8.26 (1H, s, 2-H), 8.23 (1H, s, 8-H), 6.08 (2H, s, -NH_2_), 5.93 (1H, s, 1′-H), 4.62 (1H, s, 2′-H), 4.54 (1H, s, 4′-H), 3.97–3.66 (2H, ddd, 5′-CH_2_), 2.30–2.03 (2H, ddd, 3′-CH_2_), 0.80–0.01 (15H, m, -CH_3_); ^13^C-NMR (125 MHz, CDCl_3_): δ 155.30, 152.30, 148.77, 138.96, 120.14, 92.67, 81.71, 65.64, 63.92, 32.38, 30.61, 25.97, 19.26, 18.51, 13.69. White powder; yield 92.9%; mp 256–258 °C; [α${]}_{D}^{25}$ −50 (0.1, MeOH).

#### 4.3.2. 5′-O-(Tert-butyldimethylsilyl)-6-thiophenecarboxamide-3′-deoxya-denosine (**3a**)

^1^H-NMR (500 MHz, CDCl_3_) δ: 9.30 (1H, s, -NH), 8.65 (1H, s, 2-H), 8.37 (1H, s, 8-H), 7.76–7.00 (3H, dd, 6-CH=), 6.30 (1H, s, 1′-H), 5.75 (1H, d, 2′-H), 4.50 (1H, dt, 4′-H), 4.00–3.71 (2H, ddd, 5′-CH_2_), 2.64–2.18 (2H, ddd, 3′-CH_2_), 0.88–0.00 (15H, m, -CH_3_); ^13^C-NMR (125 MHz, CDCl_3_): δ161.13, 141.39, 134.50, 133.53, 132.62, 132.42, 130.90, 130.10, 128.82, 127.91, 89.27, 81.65, 78.98, 65.57, 63.65, 31.69, 30.53, 26.02, 19.17, 18.52, 13.75. Light yellow oily liquid; yield 92.3%; mp 263–267 °C; [α${]}_{D}^{25}$ −60 (0.1, MeOH).

#### 4.3.3. 5′-O-(Tert-butyldimethylsilyl)-6-furancarboxamide-3′-deoxyadenosine (**3b**)

^1^H-NMR (500 MHz, CDCl_3_) δ: 9.40 (1H, s, -NH), 8.70 (1H, s, 2-H), 8.40 (1H, s, 8-H), 7.60–7.17 (3H, d, 6-CH=), 6.30 (1H, s, 1′-H), 5.79 (1H, d, 2′-H), 4.51 (1H, dt, 4′-H), 4.00–3.70 (2H, ddd, 5′-CH_2_), 2.69–2.18 (2H, ddd, 3′-CH_2_), 0.86–0.00 (15H, m, -CH_3_);^13^C-NMR (125 MHz, CDCl_3_): 157.51, 147.00, 145.28, 143.73, 141.66, 123.20, 117.10, 112.90, 112.19, 89.22, 81.54, 78.67, 63.63, 53.49, 31.67, 30.54, 25.95, 19.15, 18.51, 13.72. Light yellow oily liquid; yield 95.2%; mp 235–236 °C; [α${]}_{D}^{25}$ −65 (0.1, MeOH).

#### 4.3.4. 5′-O-(Tert-butyldimethylsilyl)-6-6-chloroformamide-3′-deoxya-denosine (**3c**)

^1^H-NMR (500 MHz, CDCl_3_) δ: ^1^H-NMR (500 MHz, CDCl_3_): 10.03 (1H, s, -NH), 8.93 (1H, s, 2-H), 8.45 (1H, s, 8-H), 8.18–7.23 (3H, d, 6-CH=), 6.33 (1H, s, 1′-H), 5.81 (1H, s, 2′-H), 4.53 (1H, s, 4′-H), 4.03–3.73 (2H, ddd, 5′-CH_2_), 2.66–2.22 (2H, ddd, 3′-CH_2_), 0.81–0.01 (15H, m, -CH_3_); ^13^C-NMR (125 MHz, CDCl_3_): 167.71, 163.43, 156.34, 151.24, 139.71, 132.25, 130.86, 128.80, 124.43, 124.20, 89.11, 81.61, 79.44, 63.61, 53.49, 31.43, 30.57, 25.95, 19.08, 18.49, 13.72. Yellow oily liquid; yield 85.5%; mp 231–233 °C; [α${]}_{D}^{25}$ −64 (0.1, MeOH).

#### 4.3.5. 6-Thiophenecarboxamide-3′-deoxyadenosine (**4a**)

IR (KBr) *υ*_max_: 3475, 3400, 3145, 2355, 1908, 1714, 1660, 1422, 1286, 1085, 995, 730, 639 cm^−1^; ^1^H-NMR (500 MHz, CDCl_3_) δ: 8.65 (1H, s, 2-H), 8.24 (1H, s, 8-H), 7.80–7.00 (3H, dd, 6-CH=), 6.14 (1H, s, 1′-H), 5.73 (1H, d, 2′-H), 4.58 (1H, t, 4′-H), 4.07–3.66 (2H, ddd, 5′-CH_2_), 2.91–2.23 (1H, ddd, 3′-CH_2_); ^13^C-NMR (125 MHz, CDCl_3_): δ161.38, 152.28, 150.83, 149.64, 142.33, 132.16, 130.46, 128.10, 128.05, 123.50, 91.06, 82.03, 78.89, 62.82, 31.23. MS *m*/*z* 362.1094 [M+H]^+^; white powder; yield 83.5%; mp 267–269 °C; [α${]}_{D}^{25}$−71 (0.1, MeOH).

#### 4.3.6. 6-Furancarboxamide-3′-deoxyadenosine (**4b**)

IR (KBr) *υ*_max_: 3620, 3118, 2335, 2363, 1915, 1801, 1400, 1155, 1050, 977, 835, 672 cm^−1^; ^1^H-NMR (500 MHz, CDCl_3_) δ: 8.80 (1H, s, 2-H), 8.28 (1H, s, 8-H), 7.62–7.25 (3H, d, 6-CH=), 6.20 (1H, s, 1′-H), 5.82 (1H, dt, 2′-H), 4.67 (1H, t, 4′-H), 3.75–4.18 (2H, ddd, 5′-CH_2_), 2.30–3.08 (1H, ddd, 3′-CH_2_); ^13^C-NMR (125 MHz, CDCl_3_): δ157.8, 154.53, 152.52, 150.72, 149.42, 119.42, 117.44, 113.01, 112.21, 123.65, 91.34, 81.97, 78.70, 63.00, 31.21. MS *m*/*z* 346.1324 [M+H]^+^; white powder; yield 67.2%; mp 213–215 °C; [α${]}_{D}^{25}$ −73 (0.1, MeOH).

#### 4.3.7. 6-6-Chloronicotinamide-3′-deoxyadenosine (**4c**)

IR (KBr) *υ*_max_: 3400, 3330, 3220, 2949, 1736, 165, 1605, 1298, 1135, 1094, 760, 646, 521 cm^−1^; ^1^H-NMR (500 MHz, DMSO-d6): 11.57 (1H, s, -NH_2_), 9.00 (1H, s, 2-H), 8.77 (1H, s, 8-H), δ 8.77–7.25 (3H, d, 6-CH=), 6.04 (1H, s, 1′-H), 5.78 (1H, d, 2′-OH), 5.10 (1H, t, 5′-OH), 4.65 (1H, s, 2′-H), 4.41 (1H, d, 4′-H), 3.75–3.54 (2H, ddd, 5′-CH_2_), 1.96–1.93 (1H, ddd, 3′-CH_2_); ^13^C-NMR (125 MHz, DMSO-d6): δ163.82, 153.95, 152.35, 152.03, 150.54, 150.00, 143.28, 140.22, 129.13, 125.92, 124.74, 91.38, 81.67, 75.34, 62.8, 34.24. MS *m*/*z* 391.1080 [M+H]^+^; white powder; yield 87.6%; mp 225–227 °C;[α${]}_{D}^{25}$ −74 (0.1, MeOH).

### 4.4. Cell Culture

MCF7, HepG2, SGC-7901 and MCF-10A were purchased from the National Infrastructure of Cell Line Resources in the Chinese Academy of Sciences (Shanghai Institute of Biochemistry and Cell Biology, Shanghai, China). Cells were maintained using RPMI-1640 medium with 10% fetal bovine serum. The cells were maintained at 37 °C in a 5% CO_2_-containing incubator for all treatments. Logarithmic growth phase cells were used in all experiments.

### 4.5. Cell Proliferation and Cytotoxicity Assays

The effect of compounds on the proliferation of cancer cells was determined with an MTT assay [27,28,29]. Cells monolayer in exponential growth were seeded in 96-well plates at 5 × 10^3^ cells/well. After cultured at 37 °C for 24 h, the culture medium was removed, then compounds (**3a**–**4c**) (1.25, 2.5, 5, 10, 20, 40, 80 μM) and HCPT (4, 8, 16 μM) were added. After 48 h incubation, 100 µL of MTT was added to each well and then cultured for 4 h in a 37 °C incubator. After that, the medium was aspirated, 150 µL of DMSO was then added to each well, and the 96-well plates were placed on an orbital shaker for 12 min until the formazan dissolved completely. The absorbance was measured at a test wavelength of 490 nm with a microplate reader, the optical density (OD) values were tested and the inhibitory rate and half inhibitory concentration (IC_50_) were calculated for each administration group.

### 4.6. Hoechst 33258 Staining Assay

Cells were cultured in the medium for 24 h and treated with compounds, cordycepin and HCPT. Cells were observed under inverted light microscopy (Olympus Corporation Tokyo, Japan). Then, cells were fixed for 1 h at room temperature with 4% paraformaldehyde, and Hoechst 33258 was used to stain the cells. Fluorescence microscopy (ECLIPSE Ti2; Nikon, Tokyo, Japan) was used to observe the apoptotic cells.

### 4.7. Mitochondrial Membrane Potential Assay

MCF7 cells were cultured in 6-well plates, after treatment with compounds in different concentrations and positive control group (HCPT), cells were harvested, washed with PBS, and then stained with rhodamine 123 (100 µg) for 30 min at 37 °C. The cells were collected by pipetting, washed twice with PBS and analyzed by flow cytometry (BD Biosciences, San Jose, CA, USA).

### 4.8. Cell Cycle Detection

Cells were cultured in the medium for 24 h and treated with compounds in different concentrations and HCPT incubated for 48 h. The cells were centrifuged at 1000 rpm for 10 min and washed with PBS. Subsequently, the cells were fixed with 70% ethanol for 24 h and then stained with annexin V-FITC (10 μL) and propidium iodide (PI; 10 μL). The proportion of apoptotic cells was then measured by flow cytometry.

After the treatments, the harvested cells were incubated at 70% in ice-ethanol overnight. Subsequently, they were washed twice using PBS and incubated with propidium iodide (PI) and RNase A. Finally, the proportions of cells in different cell cycle phases were determined by flow cytometry.

### 4.9. Western Blot Assay

Cells were cultured in the medium for 24 h and treated with compounds in different concentrations and HCPT for 48 h. After lysis buffer (RIPA: PMSF = 100:1) was used to lyse cells. The lysate was further sonicated and centrifuged at 12,000 rpm for 15 min, then the supernatant obtained was collected and the denatured protein mixture was subjected to SDS gel electrophoresis and transferred to nitrocellulose membranes, and blocked with containing 5% nonfat milk. Then, the membranes were incubated with the primary antibodies (1:1000, overnight 4 °C) and secondary antibodies (1:5000, 1 h). Then, ECL Solution was used to detect the antigen–antibody complexes. The data were analyzed by NIH Image J 1.53a software.

### 4.10. Statistical Analysis

Data were presented as mean ± SEM. The statistical significance was tested using Student’s *t*-test, one-way ANOVA, or two-way ANOVA with Bonferroni posttests. *p* < 0.05 was considered to be statistically significant. Tested significance is displayed in the figures as * *p* < 0.05; ** *p* < 0.01; *** *p* < 0.001.

## Figures and Tables

**Table 1:** Inhibitory effect of compounds on MCF7, HepG2, SGC-7901 cells ($\bar{x}$ ± s, *n* = 6).

Compounds	R1	R2	IC_50_ ^[a]^ (μM)		
			MCF7	HepG2	SGC-7901
Cordycepin	-OH	-H	46.85 ± 1.62	51.84 ± 1.36	51.27 ± 3.77
**3a**	-OTBDMS	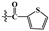	114.08 ± 2.03 **	133.75 ± 5.12 **	102.03 ± 1.08 *
**3b**	-OTBDMS	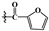	98.03 ± 0.04 **	60.43 ± 1.15 **	41.74 ± 2.36 **
**3c**	-OTBDMS	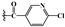	45.79 ± 1.01 **	87.01 ± 0.04 **	82.61 ± 1.48 **
**4a**	-OH	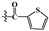	27.57 ± 0.52 ***	68.79 ± 3.34 **	38.93 ± 0.06 *
**4b**	-OH	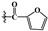	40.94 ± 1.67 **	33.37 ± 0.08 **	86.31 ± 2.35 **
**4c**	-OH	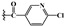	45.26 ± 0.58 **	48.39 ± 0.79 **	80.39 ± 1.05 **
10-hydroxycamptothecin (HCPT) ^[b]^			7.56 ± 0.05 ***	6.56 ± 0.45 **	7.96 ± 0.65 ***

^[a]^ All compounds were examined in a set of experiments repeated three times; IC_50_ values of compounds represent the concentration that caused 50% enzyme activity loss. ^[b]^ HCPT is positive control. compared with Cordycepin: * *p* < 0.05; ** *p* < 0.01; *** *p* < 0.001.

## Data Availability

Data are contained within the article.
